# Long-Term Dynamics of *Coxiella burnetii* in Farmed Red Deer (*Cervus elaphus*)

**DOI:** 10.3389/fvets.2015.00074

**Published:** 2015-12-11

**Authors:** David González-Barrio, Isabel G. Fernández-de-Mera, José Antonio Ortiz, João Queirós, Francisco Ruiz-Fons

**Affiliations:** ^1^Health and Biotechnology (SaBio) Group, Spanish Wildlife Research Institute IREC (CSIC, Universidad de Castilla–La Mancha, Junta de Comunidades de Castilla–La Mancha), Ciudad Real, Spain; ^2^Medianilla S. A., Benalup-Casas Viejas, Spain; ^3^Centro de Investigacão em Biodiversidade e Recursos Genéticos (CIBIO), InBio Laboratório Associado, Universidade do Porto, Vairão, Portugal; ^4^Departamento de Biologia, Faculdade de Ciências da Universidade do Porto, Porto, Portugal

**Keywords:** control, epidemiology, Q fever, wildlife, zoonosis

## Abstract

Several aspects of the dynamics of *Coxiella burnetii* that are relevant for the implementation of control strategies in ruminant herds with endemic Q fever are unknown. We designed a longitudinal study to monitor the dynamics of exposure to *C. burnetii* in a red deer herd with endemic infection in order to allow the design of Q fever-specific control approaches. Other relevant aspects of the dynamics of *C. burnetii* – the effect of herd immune status, age, season, and early infection on exposure, the average half-life of antibodies, the presence and duration of maternal humoral immunity, and the age of first exposure – were analyzed. The dynamics of *C. burnetii* in deer herds seems to be modulated by host herd and host individual factors and by particular host life-history traits. Red deer females become exposed to *C. burnetii* at the beginning of their second year since maternal antibodies protect them after birth and during the main pathogen shedding season – at the end of spring-early summer. Infection pressure varies between years, probably associated with herd immunity effects, determining inter-annual variation in the risk of exposure. These results suggest that any strategy applied to control *C. burnetii* in deer herds should be designed to induce immunity in their first year of life immediately after losing maternal antibodies. The short average life of *C. burnetii* antibodies suggests that any protection based on humoral immunity would require re-vaccination every 6 months.

## Introduction

*Coxiella burnetii* is a worldwide-distributed Gram-negative intracellular bacterium that causes Q fever, a zoonotic disease shared by humans and animals. Infection with *C. burnetii* in humans is usually asymptomatic but it may trigger acute and chronic clinical manifestations. *C. burnetii* is also one of main pathogens causing reproductive losses in livestock (1) and reproductive failure in pets (2, 3) and wildlife (4–7). Clinical signs of Q fever in domestic ruminants are diverse; it has been associated with sporadic cases of abortion, premature delivery, stillbirth, and weak offspring in cattle, sheep, and goats, but epidemics with increased reproductive failure have been reported for sheep and goats mainly (8). Since *C. burnetii* infection does not always manifest clinically, the extent of *C. burnetii* infection in animals is probably underestimated.

Exposure to *C. burnetii* is increasingly reported in wildlife, e.g., (i) white-tailed deer – *Odocoileus virginianus* – in the eastern US (9); (ii) rats – *Rattus norvegicus* and *Rattus rattus* – in the UK (10) and the Netherlands (11); or (iii) European rabbits – *Oryctolagus cuniculus* – and Eurasian wild boar – *Sus scrofa* – in the Iberian Peninsula (12–14). Recently, González-Barrio et al. (15) found that *C. burnetii* circulates endemically in Iberian red deer populations. This study suggests that the red deer plays an important role in the maintenance of *C. burnetii* in Europe. Thus, the analysis of the dynamics of *C. burnetii* in red deer may be of interest to prevent Q fever transmission at the wildlife–livestock–human interface (16). Furthermore, the presence of *C. burnetii* in red deer may have implications for red deer itself and coexisting wild species (4, 7, 17, 18). Q fever may be an important cause of reproductive losses in red deer farming (7), an activity that is increasing worldwide (19). Therefore, deer producers could be interested in implementing Q fever prevention and control measures that would benefit from knowledge of the effect of deer farming particularities on *C. burnetii* dynamics.

Information on the dynamics of *C. burnetii* in endemic ruminant herds and on driving factors (host population, host individual, and environmental) is scarce. A trade-off between infection pressure and herd immunity may influence infection dynamics in *C. burnetii* endemic herds, which may modulate the efficiency of vaccination trials. Recently, it has been postulated that in endemic dairy cattle herds the immune status of the population drives exposure to *C. burnetii* (20). According to this postulate, high levels of protection in an endemic herd may lead to a reduction in environmental contamination with *C. burnetii*, therefore reducing transmission. However, as long as the immune status of the herd changes with time (i.e., herd immunity decreases due to culling of immune individuals) while *C. burnetii* persists in latently infected animals or in infected fomites (21), the circulation of *C. burnetii* reactivates and expands within the population. Currently, no long time series study has demonstrated that the immune status of a *C. burnetii* endemic ruminant population changes with time to support this postulate. Information from long time series would provide a significant boost to understand the epidemiology of Q fever and plan any prevention and/or control approach.

Apart from host population factors, host individual factors (e.g., age, maternal-derived immunity or acquired immunity, among others) may modulate the dynamics of *C. burnetii* (14). Currently, the presence, prevalence, and duration of maternal anti-*C. burnetii* antibodies and their effect in the outcome of natural exposure to *C. burnetii* are poorly understood. If ­vaccination of animals at early ages (before natural infection by *C. burnetii* takes place) is to be performed (22), knowledge on the exact timing for vaccination – i.e., the time at which maternal antibodies disappear and prior to exposure to *C. burnetii* – could be paramount to warrant protection. In dairy cattle, the transmission of colostral antibodies to calves borne from seropositive cows has been reported (23) but the duration of these antibodies was not monitored. Could early exposure to *C. burnetii* of individuals in endemic herds modulate infection with the individual’s age? The effect of early exposure to *C. burnetii* on future protection against infection is also poorly known. In natural infections in domestic ruminant females, a non-immune animal is supposed to become infected and undergo a primary subclinical infection at early ages (24) that reactivates during the first pregnancy. Understanding the effect of natural early exposure to *C. burnetii* on future exposure would perhaps allow predicting the effect of vaccination at early ages on protection against *C. burnetii* infection. The likelihood of becoming infected by *C. burnetii* increases with age (25). Indeed, age-related *C. burnetii* serological patterns have been reported in domestic ruminants (26, 27) with highest seroprevalence in cows and sheep aged 3–5 years. Is this pattern similar in farmed red deer?

In this study, we aimed to answer different questions that are relevant to understand *C. burnetii* dynamics in endemic ruminant herds in the long time scale and that are, therefore, essential for the efficient planning and application of any Q fever control measure, such as vaccination. The following objectives were addressed in the study: (1) analysis of long-time variation in exposure to *C. burnetii*; (2) determination of the effect of herd immune status on the risk of exposure to *C. burnetii* of yearling females; (3) test of the effect of deer age on exposure and humoral response to *C. burnetii*; (4) study of the effect of deer life-history traits (i.e., concentrated calving) on exposure to *C. burnetii*; (5) investigation of the effect of natural exposure to *C. burnetii* at early ages over the future dynamics of exposure; (6) determination of the average half-life of antibodies against *C. burnetii*; (7) estimation of the presence, prevalence, and duration of maternal antibodies; and (8) determination of the age at which deer get exposed to *C. burnetii* for the first time in their life. To achieve our objectives, a *C. burnetii* endemic red deer herd was selected as model. Therefore, the present study provides a case report on the dynamics of *C. burnetii* in a red deer farm with a history of *C. burnetii* infection in humans and reproductive failure in deer.

## Materials and Methods

### Study Farm and Management Schemes

The study was performed in a semi-extensive red deer farm located in the province of Cádiz (Southern Spain) that was found consistently positive to *C. burnetii* in consecutive studies (7, 28). The number of deer on the farm was approximately 500 females and 80 males along the study period, with only slight inter-annual variations in the number of reared animals. The deer are semi-extensively bred within large (6–8 has) enclosures separated by high-wire fencing in batches of approximately 60–80 females; males are kept in separate enclosures. The habitat in the deer enclosures consists on patches of natural Mediterranean scrubland – mainly composed by evergreen (*Quercus ilex*) and cork (*Quercus suber*) oak trees – with large areas of year-round irrigated prairies. Animals are bred in separate but contiguous batches according to sex and breeding status. Artificial feed is provided daily on the farm.

The deer are managed from two to four times per year at maximum to avoid excessive stress. Management is carried out for sanitary issues, weaning, reposition, and artificial insemination. Hinds give birth naturally – i.e., without human intervention – in hidden areas of scrubland patches within enclosures from the end of April to the beginning of May. Calves are weaned in August at 3.5 months of age and thereafter they are kept in male and female batches in separate enclosures. Ear tagging is performed at weaning for identification of individuals. Calves are managed again at 7 months of age for sanitary reasons. When animals are 13 months old, a selection of yearling females and males for farm reposition is performed and the rest of yearlings are sold. Reposition yearling females are inseminated at the age of 16 months when they join other hinds in existing batches of reproductive females. Selected yearling males are kept separate from stags in reproductive condition. Adult deer, both males and females, are managed for sanitary control in January and in August each year. Reproductive females (>16 months old) are annually managed for artificial insemination in September.

Reproductive hinds in the study farm are culled annually according to their reproductive fitness or health status. Average productive life of deer females in the farm is unknown; some hinds remain productive for 13–15 years but most are culled at 4–5 years of life.

Management schemes in the farm are scheduled to carry out batch, reproductive, and sanitary management issues without inducing excessive stress in the deer that, although farm-bred, still behave like wild animals. Therefore, animal sampling could only be performed according to the management schedule of the farm except for the monitoring of antibodies in the 2013 cohort (see details in the following section).

### Survey Design

We designed a retrospective survey to search for the presence and the level of antibodies against *C. burnetii* in deer sera collected in the study farm for disease surveillance purposes. The retrospective survey was aimed at testing for variation in herd seroprevalence over time (Objective 1), the effect of herd immune status on the risk of exposure of yearling females (Objective 2), age-related variation in immune status of individual deer (Objective 3), seasonal variation in infection risk (Objective 4), influence of exposure at early ages on future exposure to *C. burnetii* (Objective 5) and the average half-life of *C. burnetii* antibodies (Objective 6). Blood samples were collected according to farm disease surveillance schemes and in the framework of Spanish and EU laws for notifiable disease surveillance. Therefore, no Animal Ethics Committee approval was required for the collection of blood samples from deer for the retrospective study.

To determine the inter-annual variation in exposure to *C. burnetii*, we carried out a selection of deer sera (yearling, 12–24 months old, and adult, >24 month-old females; *n* = 1021) collected in the farm along 12 consecutive years (2003–2014). Minimum annual sample size was estimated with WinEpi (http://winepi.net/sp/index.htm). The estimated sample size for each age-class – yearling and adult – with a 95% confidence level for a minimum seroprevalence of 5% with an accepted 10% error was 19; this minimum sample size was covered each year for each deer age-class. Selection of sera was carried out according to batch origin to obtain a balanced subset of samples that provides representative information of the real status of *C. burnetii* prevalence in the herd. For homogeneity of results, only sera collected in summer were selected to estimate inter-annual variation in the humoral immune status of deer in the farm.

To test for the rest of aforementioned objectives (2–6), we selected sera from reposition females belonging to three different cohorts – animals born in 2008, 2009, and 2010. Blood was collected from these females at least in four consecutive occasions (i.e., from 7 to 27 months old) and up to 13 consecutive occasions (i.e., from 7 to 78 months old). Deer were surveyed in summer and winter each year. Serum samples were obtained for the 2008 cohort at 7, 13, 20, 27, 32, 38, 44, 51, 56, 62, 67, 73, and 78 months of life. The same survey was carried out for the 2009 cohort but up to 67 months of life and the 2010 cohort could be surveyed up to the 56th month of life. Minimum sampling size at each survey time was estimated with WinEpi employing the same parameters described above. Although culling with age reduced the number of available samples with individuals’ age, sampling size was above 19 for each month class except for individuals at 73 and 78 months of age (*n* = 13 in both cases).

Finally, to test for the presence, prevalence, and duration of maternal antibodies (Objective 7) and for the age at which deer get exposed to *C. burnetii* for their first time in life (Objective 8), serum samples from 21 calves born in 2013 were prospectively collected at 2, 3, 7, 13, 14, 19, and 20 months of life. This subset of the 2013 cohort was specifically surveyed to achieve these objectives since blood from calves in the farm is normally first collected at 7 months of age. The Research Ethics Commission of Castilla – La Mancha University Animal Ethics Committee (Spain) approved this research.

Since we initiated an experimental vaccination trial in the study herd in January 2012 with a *C. burnetii* phase I inactivated vaccine, animals that were included in this study for 2012, 2013, and 2014 were exclusively unvaccinated animals of the control group. The interpretation of the evolution of the immune status of the herd will be carried out in this study considering this potential confounding factor for 2012–2014 results.

### Serological Analyses

Serology has been widely employed to test for the status of *C. burnetii* in ruminant populations (25, 28) and to understand its dynamics even though a proportion of infected individuals may not seroconvert (29). In a vaccination trial in the study, deer farm (the authors, unpublished) near the 90% of vaccinated seronegative deer seroconverted after a single dose of a *C. burnetii* inactivated phase I vaccine. This percentage was close to the 100% after a boosting dose 3 weeks later. Therefore, in contrast to domestic ruminants (e.g., sheep, 22), it is expected that a high percentage of infected red deer display detectable levels of anti-*C. burnetii* antibodies in ELISA. Therefore, ELISA was employed to study the dynamics of infection by *C. burnetii*.

Blood was collected from the jugular vein into sterile tubes and it was thereafter kept at 4°C and transported to the laboratory. Blood was centrifuged at 3,000 × *g* for 10 min and the serum obtained was preserved at −20°C until analyses were performed. The presence of specific antibodies against *C. burnetii* phase I and II antigens in deer sera was determined with a commercial indirect ELISA test (LSIVet™ Ruminant Q Fever Serum/Milk ELISA Kit, Life Technologies, Carlsbad, CA, USA) with an in-house modification in the secondary antibody (Protein G−Horseradish peroxidase, Sigma-Aldrich, St. Louis, MO, USA) that had been validated for wild ungulates (15). ELISA results were expressed as the sample-to-positive control ratio (SP). For each sample, the SP was calculated according to the formula:

$$\text{SP}=\frac{\text{(ODs}-\text{ODnc)}}{\text{(ODpc}-\text{ODnc)}}\times \text{100}$$

where “ODs” is the optical density of the sample at a dual wavelength 450–620 nm, “ODnc” is the optical density of the negative control, and “ODpc” is the optical density of the positive control. All SP values ≤40 were considered as negative, whereas SP values >40 were considered as positive. The SP ratio was considered as a proxy of the level of antibodies against *C. burnetii* as suggested by the manufacturer.

### Statistical Analyses

Statistical analyses were carried out to test different hypotheses: (i) the immune status of the herd is negatively related to the incidence of infection by *C. burnetiii* in yearling females (Objective 2); (ii) the age of individuals is positively related to seroprevalence and antibody levels (Objective 3); (iii) there are seasonal variations in the rate of exposure to *C. burnetii* (Objective 4); and (iv) variations in the immune status at early ages modulates future exposure to *C. burnetii* (Objective 5).

To assess for the effect of herd immune status on the incidence of *C. burnetii* in yearling females, we performed Spearman correlations with the annual incidence (presence/absence of *C. burnetii* antibodies) in yearlings as response variable and three different explanatory variables that were tested separately: (i) seroprevalence in adult females in the same year (t); (ii) seroprevalence in adult females in the previous year (t−1); and (iii) average seroprevalence in adult females in years t, t−1, and t−2 (2 years before). Seroprevalence in adult females was employed as a proxy of herd seroprevalence because adult females constitute around the 75% of the herd. Spearman correlations were also employed to test the relationship between antibody levels and individuals’ age.

Mann–Whitney *U* non-parametric tests for independent samples were employed to test for the alternative hypothesis of statistically significant differences in average antibody levels by season. Chi-square tests were employed for the same purpose with seroprevalence as response variable.

Finally, the influence of the immune status of individuals at early ages (7 months old; presence/absence of *C. burnetii* antibodies) on the evolution of the humoral immune response against *C. burnetii* infection along individual’s age was tested by repeated measures ANOVA. Individual squared SP values were transformed into natural logarithms for normality and were employed as response variable. For this analysis, we used data obtained from the 2008, 2009, and 2010 deer cohorts.

IBM SPPS v22.0 (IBM, Armonk, NY, USA) was employed for statistical analyses. Exact Clopper–Pearson 95% confidence intervals (95%CI) were estimated for prevalence values using Quantitative Parasitology 3.0 software (http://www.zoologia.hu/qp/qp.html).

## Results

**Figure 1:**
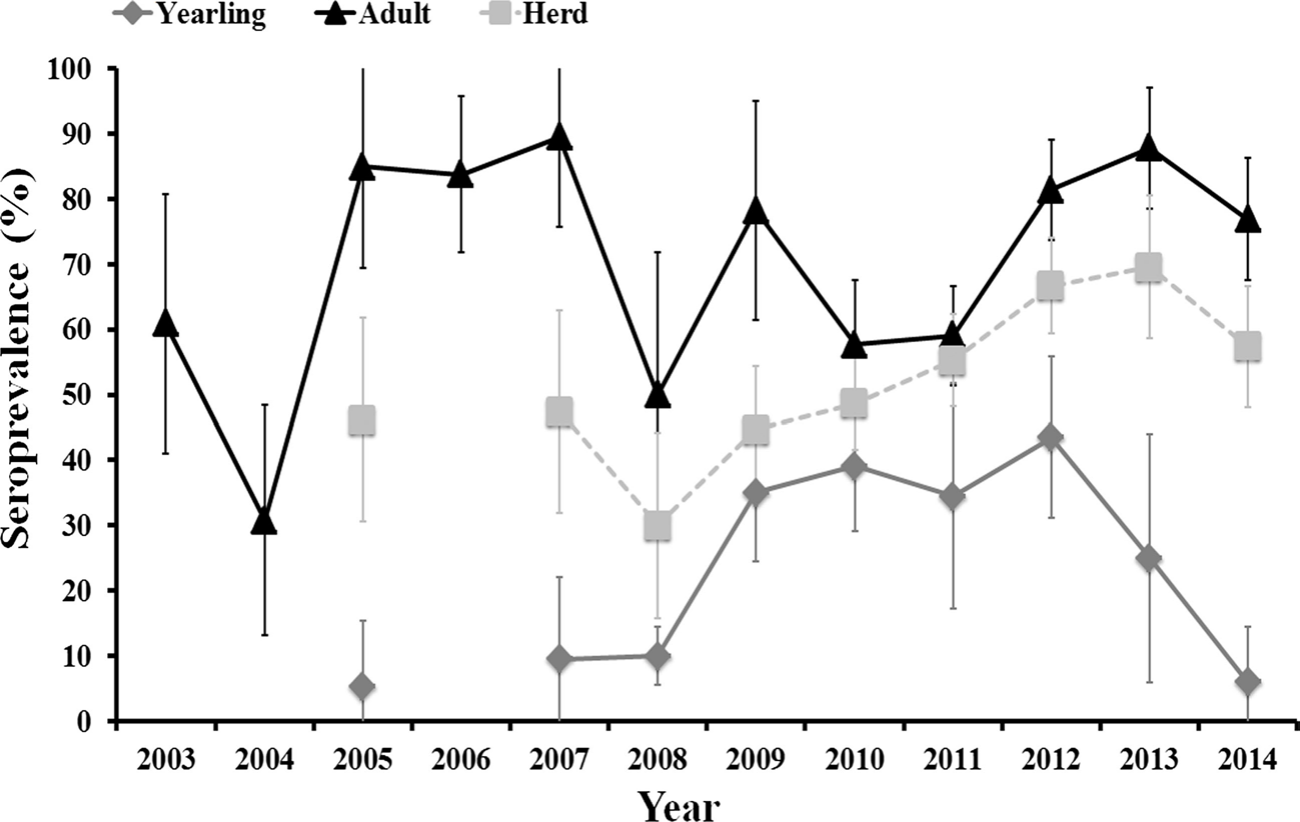
**Evolution of herd (yearling + adult) average and age-class-specific (yearling, 12–24 months old, and adult, >24 months old) seroprevalence (and associated 95% exact confidence intervals) from 2003 to 2014**.

A total of 373 (inter-annual sampling size range: 19–92) and 648 (inter-annual sampling size range: 19–159) serum samples collected in summer from yearling and adult females, respectively, were selected for the cross-sectional approach during 2003–2014. The inter-annual evolution of herd (yearling + adult) and age-class-specific seroprevalence from 2003 to 2014 is shown in Figure 1.

Average annual herd and age-specific seroprevalence values varied between years. There was no clear time scale pattern in inter-annual herd seroprevalence that remained above 30% and below 70% along the study period. Seroprevalence in adult females fluctuated between years with periods of 1–3 consecutive years of high values (above 70%) followed by periods of 1–2 consecutive years of medium values (from 30 to 60%). The high seroprevalence in unvaccinated females in 2012, 2013, and 2014, even though >70% of the herd had been vaccinated (the authors, unpublished), indicate a natural period of high herd humoral immunity similar to that observed in 2005–2007. A unimodal pattern was observed in yearlings. The observed age-related pattern of humoral immunity in individual deer shows that we can assimilate annual seroprevalence in yearlings to annual incidence ratio. Incidence in yearlings was low (5–10%) from 2005 to 2008 (although samples were not analyzed in 2006), it increased (35–43%) from 2008 to 2012, and it steeply decreased (25% in 2013 and 6% in 2014) from 2012 onward.

Incidence in yearlings remained low during the periods in which high seroprevalence was observed in adult females and increased during a period in which the seroprevalence in adult females was lower than in preceding years. Incidence was nonetheless not statistically influenced by seroprevalence in adult females at times t (survey year) and t−1 (previous year to survey), and by average seroprevalence in adult females in years t to t−2. However, trends in all three relationships were negative, that is increasing incidence in yearlings related to decreasing seroprevalence in adults in the same or in previous years.

**Figure 2:**
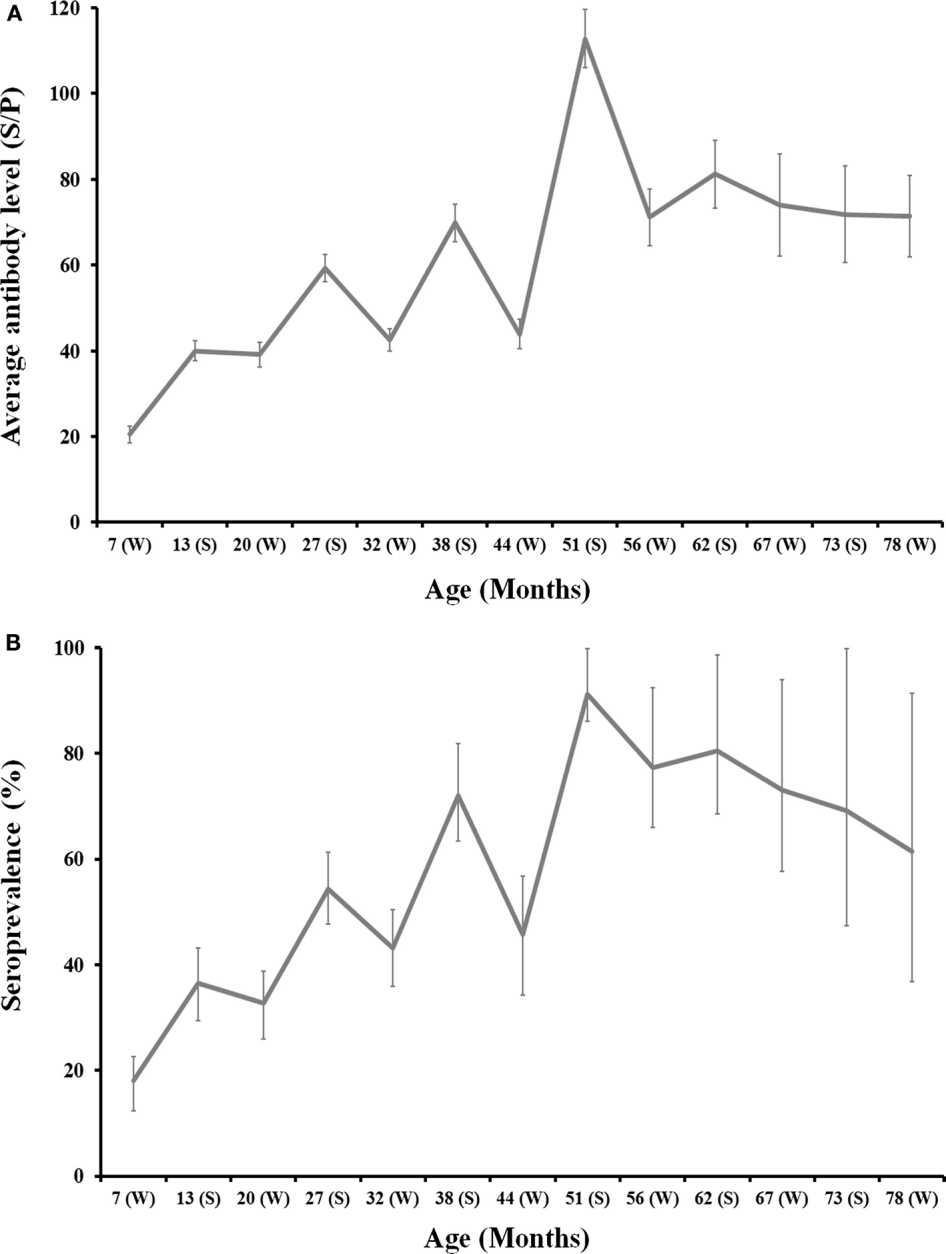
**Age-related evolution of *C. burnetii* level of antibodies (A) and seroprevalence (B) for 2008, 2009, and 2010 deer cohorts together**. Season is depicted in the *x*-axis of each chart (W: Winter; S: Summer). Exact 95% confidence intervals for seroprevalence and SE for average antibody levels are displayed in the charts.

One thousand four hundred and forty-five sera from 217 animals born in 2008 (*n* = 97), 2009 (*n* = 92), and 2010 (*n* = 28) were sequentially analyzed from 7 (calf) up to 78 (adult) months of life to estimate the evolution of antibodies against *C. burnetii* with age. Not every animal could be surveyed sequentially along the study period because of annual culling for health or productive reasons. On average, both seroprevalence and the level of antibodies in individual deer increased with age and this pattern was evidenced in animals from the three cohorts (Figure 2). Age (in months) and the level of antibodies were statistically significantly correlated (rho = 0.416, *p* < 0.001). This result confirms increasing levels of antibodies with deer age, but highest antibody levels were observed between 4 and 5 years of life.

The monitoring of individual deer sera by ELISA showed an evident seasonal pattern in antibody levels and seroprevalence. Both the level of antibodies and seroprevalence peaked in summer each year and decreased through winter (Figure 2). Seasonal differences were statistically significant: (i) Average SP in summer was 73.3 ± 2.5 in contrast to 37.8 ± 1.4 in winter (*Z* = −12.89, df = 1, *p* < 0.001) and (ii) 67.3% (95%CI: 62.8–71.7) of animals surveyed in summer had antibodies in contrast to 36.0% (95%CI: 32.6–39.5) in winter (χ^2^ = 110.93, df = 1, *p* < 0.001). This pattern was evident for any of the three cohorts surveyed (Figure 2).

**Figure 3:**
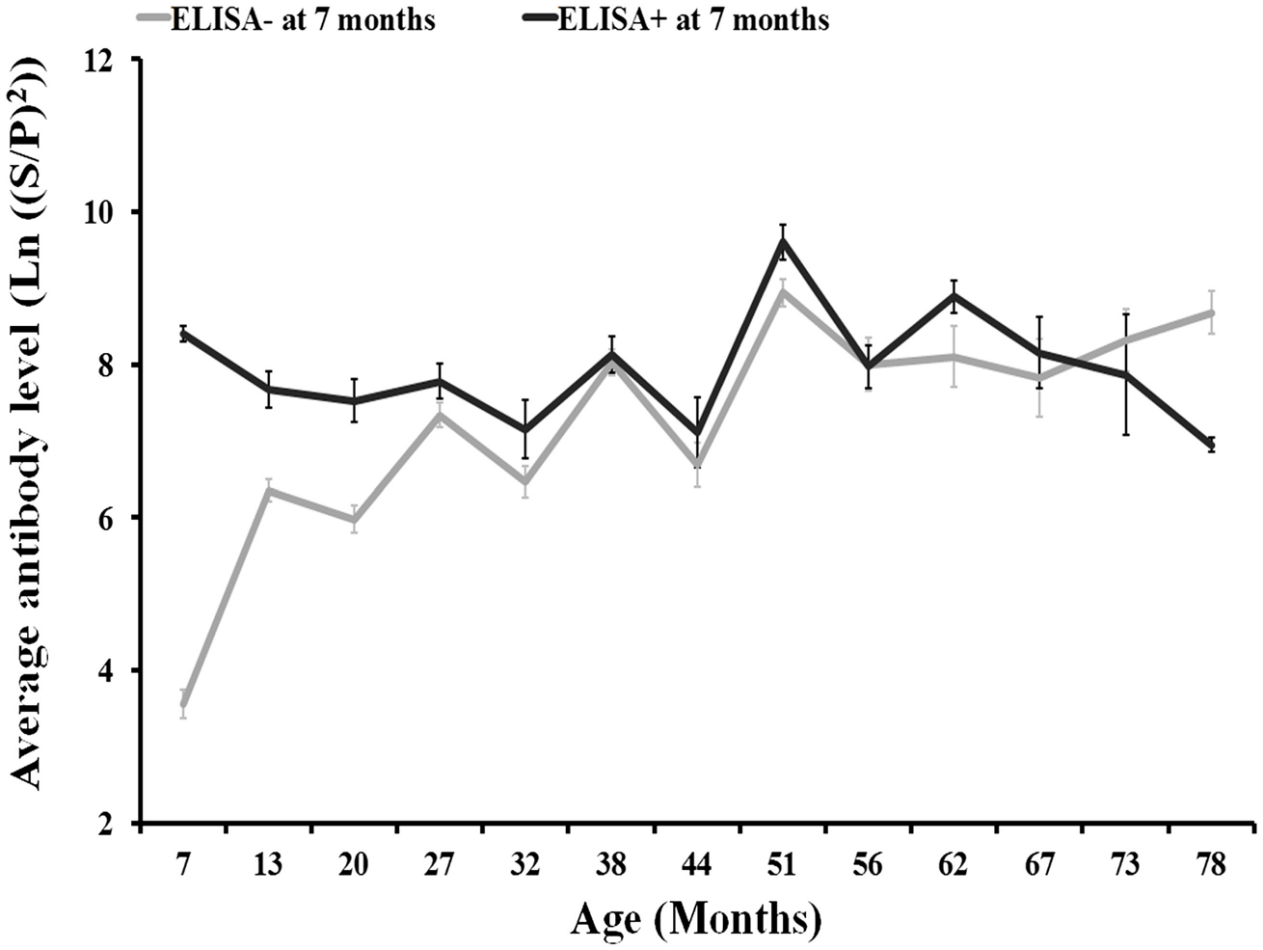
**Evolution of antibody levels (and associated SE) with individuals’ age according to the presence/absence of anti-*C. burnetii* antibodies by ELISA (SP > 40) at 7 months of age for 2008, 2009, and 2010 deer cohorts together**.

Results from the repeated measures ANOVA showed that differences in the evolution of the level of antibodies in relation to the presence/absence of *C. burnetii* antibodies at 7 months of age were not statistically significant (Figure 3). Around the 82% of individuals in the 2008–2010 cohorts were seronegative at 7 months of age.

**Figure 4:**
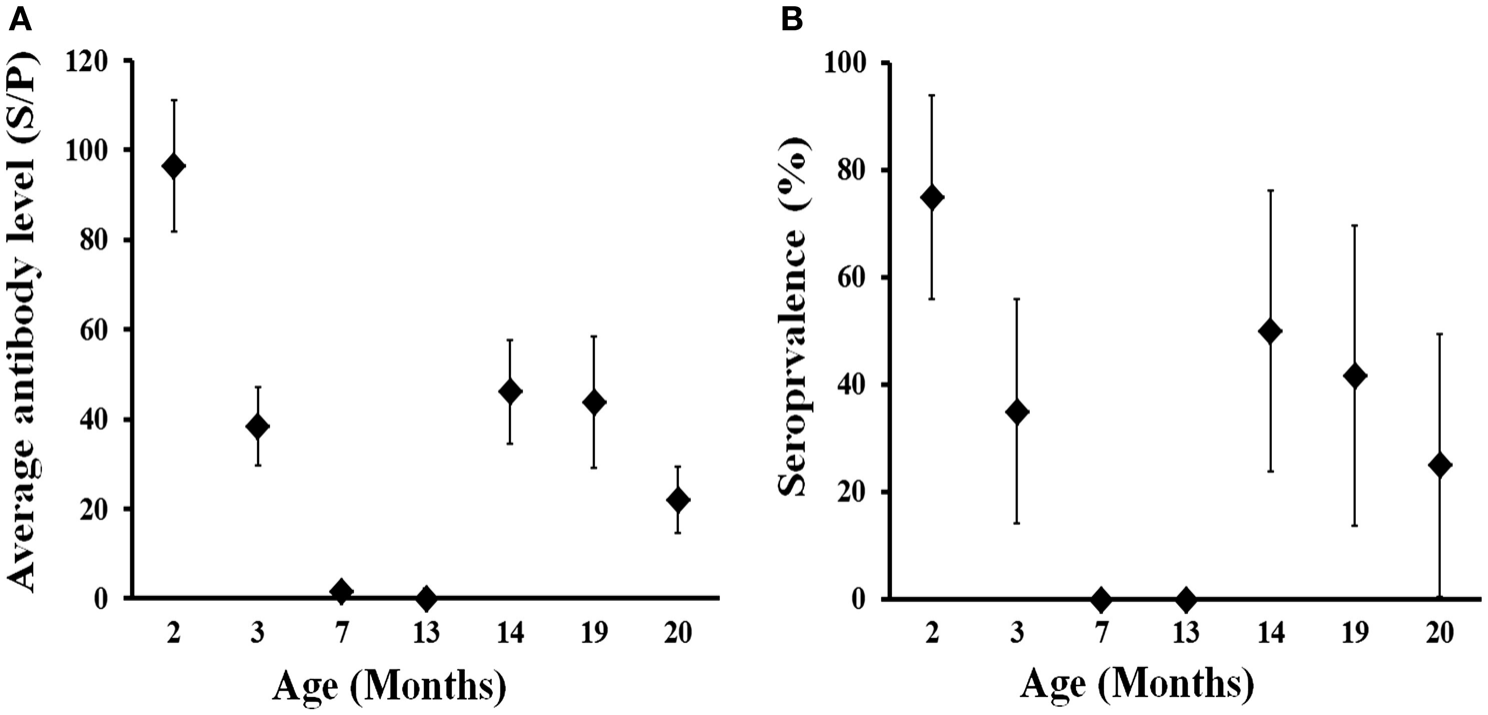
**Age-related evolution of *C. burnetii* level of specific antibodies (A) and seroprevalence (B) in farmed red deer (2013 cohort)**. Exact 95% confidence intervals for seroprevalence and SE for average antibody levels are displayed in the charts.

The evolution of the presence and level of antibodies in calves born in 2013 from 2–20 months of life are shown in Figure 4. High levels of antibodies were evidenced in calves at 2 months (June 2013), when 75% of them displayed an SP ratio  > 40 (i.e., seropositive). Thereafter, both the level of antibodies and seroprevalence decreased sharply in 1 month (July 2013) and disappeared at 7 months (November 2013). Animals remained seronegative at 13 months of age (May 2014) and then became seropositive at 14 months (June 2014), 2 months after the calving season in the farm. This seroconversion was most probably caused by natural infection and affected 50% of the animals. The average level of antibodies derived from natural infection at 14 months was lower than that acquired from their mothers during lactation (at 2 months); in seropositive animals average SP ratio was 122.4 at 2 months (*n* = 15) in comparison to 77.8 in seropositive animals at 14 months of life (*n* = 7). Both seroprevalence and antibody level remained at similar values at 19 months (November 2014) but decreased thereafter notably a month later (December 2014). This observation and the seasonal pattern observed indicate that the expected average life of antibodies against *C. burnetii* could be around 6 months. These results show that deer become exposed to *C. burnetii* for the first time in life mainly at around 12–14 months of age.

## Discussion

Understanding the factors that drive the dynamics of endemic pathogens is paramount to design and efficiently apply any preventive or control measure. Vaccination with phase I inactivated *C. burnetii* vaccines – one of the main Q fever control tools in domestic ruminants (30) – is recommended for naïve or low-prevalence herds (31) but not for endemic herds (22, 32). However, the status of *C. burnetii* in an endemic host population may present inter-annual variation (20) with years in which the percentage of naïve individuals in the population is high, followed by years in which this percentage is low. Identification of time windows with high percentage of naïve individuals in a *C. burnetii* endemic population would allow the implementation of vaccination trials. During these time windows, the percentage of susceptible individuals that could be protected by vaccination would be enhanced. Determining the factors that modulate the dynamics of *C. burnetii* in endemic populations would allow predicting the occurrence of appropriate windows to implement vaccination. This study improves our understanding of the dynamics of *C. burnetii* in endemic ruminant herds and driving factors that could allow more efficient control approaches in the future.

### Risks of Infection by *C. burnetii* Associated to Red Deer

Red deer may be a relevant reservoir of *C. burnetii* in Europe because of its increasing relevance as a game resource, its current demographic status and the status of *C. burnetii* in red deer populations. Deer farming is increasing (19) and likewise populations of free-roaming red deer currently display increasing demographic trends (33). Additionally, free-roaming deer populations are increasingly managed as extensively bred ruminants (supplementary feeding, fencing, translocations) but lacking appropriate sanitary control (34). Changes in livestock production schemes in the Netherlands – increasing number of goat herds without *C. burnetii* control – led to the 2007–2010 epidemics of human Q fever (35), demonstrating how important demographic changes of a single host may be to increase the risk of infection by *C. burnetii*. Interestingly, farmed and free-roaming Iberian red deer populations display similar population and individual seroprevalence values to livestock (15, 24). Furthermore, increasing geographic distribution and population density of red deer in Europe may increase the implication of this wild species in future Q fever epidemics. Prevention would only be possible if accurate scientific knowledge is available. Our study provides insights into poorly studied epidemiological aspects of the dynamics of *C. burnetii* in red deer populations.

### Long-Term Dynamics of *C. burnetii* in Farmed Red Deer

There is a main question to answer in relation to the dynamics of *C. burnetii*: Does the status of *C. burnetii* change with time in an endemic ruminant herd? Our results for 12 consecutive years show fluctuation in the status of *C. burnetii* prevalence in a ruminant herd in which the pathogen circulates endemically. Piñero et al. (20) also provided evidence of inter-annual variation in endemic dairy cattle herds but within a shorter time period.

Inter-annual variation of the status of an endemic pathogen in a herd could be the consequence of the trade-off between pathogen burden and host immunity if we assume that there are no changes in the composition of the herd – e.g. size, culling and import rates, age, and sex structures – and the influence of external pathogen sources remains constant (14, 20). Those features remained constant along the study period in the deer farm. Even the potential influence of other sources of *C. burnetii* such as European rabbits – *O. cuniculus* – remained similar along the study period, with *C. burnetii* seroprevalence from 2005 to 2013 above 50% (13). Therefore, herd immunity effects seem to be the most probable cause of changes in the epidemiological status of *C. burnetii* in the herd. However, in spite of the observed negative trend in the relationship between incidence in yearling females – probably associated with infection pressure – and seroprevalence in adult females, relationships were not statistically significant. Increasing incidence in yearling females from 2009 to 2012 coincided with a period of lower seroprevalence values in adult females in comparison to 2005–2007. By contrast, there was a steep decrease in incidence in yearlings from 2012 to 2014 when seroprevalence in adult (non-vaccinated) females was again high. This observation could potentially be linked to herd vaccination against *C. burnetii* (the authors, unpublished data) or alternatively be the consequence of a natural period of high humoral immunity in the herd; but in any case, it indicates variation in infection pressure. Interestingly, an outstanding rate of reproductive failure in the herd in 2011 – that was presumably caused by Q fever (7) – coincided with the increasing incidence observed in yearlings in 2009–2012.

It was unfortunately impossible to monitor the presence and burden of *C. burnetii* in the environment in the farm (aerosols, soil, water, food, pastures) along the study period in order to accurately estimate the evolution of infection pressure with time. However, this has been carried out in dairy cattle and changes in the epidemiological status of *C. burnetii* in dairy cattle herds are linked to the detection of *C. burnetii* in manure, air, and dust samples (20). According to Piñero et al. (20) high herd humoral immunity levels would reduce shedding of large burdens of *C. burnetii* to the environment, therefore, reducing infection pressure. This would result in a reduced incidence in naïve (yearling) individuals in the herd. Another observation that may support variation in infection pressure with time is that 30.9% (30/97) and 9.9% (9/91) of deer calves born in 2008 and 2009, respectively, were seropositive to *C. burnetii* at 7 months of age (naturally infected in their first year of life) in contrast to 0% (0/21) in 2013. This pattern paralleled observations in incidence in yearling females. These changes could be linked to the implementation of vaccination in the herd from 2012 onward since vaccinated and unvaccinated deer coexist in existing enclosures in the farm.

### Short-Term Herd Effects on the Dynamics of *C. burnetii* in Red Deer

Intra-annual variation in exposure to *C. burnetii* has been previously suggested in wildlife studies (36). Ruminant females shed *C. burnetii* mainly around parturition and, therefore, in species with a defined breeding season shedding should be concentrated. In contrast to dairy cattle, which breed along the year, the breeding season of the red deer is concentrated at the end of spring (37). This fact implies that, within a year, there is a predominant shedding season during which the risk of exposure of individuals is higher. The short half-life of *C. burnetii* antibodies allowed differentiating that the risk of exposure in winter is much lower than by the end of spring-early summer, which is consistent with a predominant shedding season in deer than coincides with the breeding season. This particularity of the epidemiology of *C. burnetii* in farmed red deer may favor the implementation of control strategies since adequate management measures in liaison with medical treatments can significantly reduce the exposure of individuals around the breeding season.

### Host Individual Traits Influencing *C. burnetii* Dynamics in Red Deer

Host individual traits may modulate the relationship that a host establishes with *C. burnetii* and age-related effects have been described frequently (25). In this study, we found a significant increase in seroprevalence and antibody level with the individuals’ age. This may be caused by cumulative effects of continuous exposure to *C. burnetii* with time or may be linked to increasing immune competence with the individual’s age. Two observations point to an effect of host immune competence as the causal factor for this age-related increase in seroprevalence and antibody levels: (i) the increasing trend in both parameters up to the fourth year of life (similar to findings in cattle, 27) and the decreasing pattern thereafter; and (ii) the low average half-life of anti-*C. burnetii* antibodies observed (discussed below).

Acquired immunity after natural infection by *C. burnetii* at early ages may have a protective effect over the outcome of future infections since reproductive failure caused by Q fever is more evident in primiparous females and decreases with age (22, 32). However, we observed that in calves exposed to *C. burnetii* at 7 months of age the average humoral immune response induced by infections in adulthood did not differ from that observed in non-exposed calves at that age. Whether the effect derived from natural infection is similar to what we would expect from vaccination is difficult to predict, but this finding suggests that acquired immunity at early ages does not prevent re-infection by *C. burnetii* in the future. This could be linked to the short average half-life of *C. burnetii* antibodies observed in deer. Vaccination of deer at early ages with an appropriate re-vaccination calendar would perhaps induce long-lasting protection against infection by *C. burnetii*.

The pattern of antibody levels in the 2013 cohort suggest that deer calves get antibodies from their mothers early in their lives that then disappear before their seventh month of life. Maternal-acquired antibodies have also been reported from cattle calves (23). Dairy cows infected with *C. burnetii* maintain detectable levels of antibodies along the gestation period and even after partum (38) that are transmitted to new-born calves with the colostrum. Whether maternal antibodies protect against infection by *C. burnetii* is unknown. Results from the 2013 cohort suggest that maternal antibodies protect calves in their first year of life – perhaps in association with the concentrated shedding season in late spring, but the presence of antibodies in 7-month-old animals of the 2008/2009 cohorts contradicts that observation. If we assume – on the basis of incidence rates – that infection pressure was higher in 2008/2009 than in 2013 and that a high percentage of calves born every year acquire maternal antibodies, we may hypothesize that under high infection pressure in the herd a percentage of the calves are not protected during their first year of life. Only proper experimental approaches with a controlled challenge would offer information to understand the effect of humoral immunity on protection (39). Nonetheless, our findings suggest that deer calves should be vaccinated for the first time when they are around 5–6 months of life. The exact timing for vaccination should be determined through future experiments with a higher sampling frequency.

An interesting finding that should be born in mind when planning vaccination protocols in deer farms is that any protection linked to humoral immunity would last only around 6 months. Maternal antibodies in the 2013 cohort were high at 2 months of age and completely disappeared 5 months later. This observation and the sharp decrease of antibodies from natural infection from months 14–19 to month 20 suggest an average half-life of anti-*C. burnetii* antibodies of 5–6 months without natural re-infections. Therefore, re-vaccination every 6 months would be recommendable to maintain humoral immunity in deer. The average half-life of antibodies in other species may be higher since antibodies can be detected even a year after infection in humans (40).

## Conclusion

Red deer are able to maintain *C. burnetii* and transmit it to other wildlife, livestock, pets, and humans. Current knowledge on the status of *C. burnetii* in red deer in Iberia together with results obtained in this study points to this species as a source of Q fever that needs to be considered by animal and public health authorities.

In endemic herds, *C. burnetii* inter-annual dynamics may be modulated by host herd and individual factors that should be considered for planning efficient control approaches. Particular host life-history traits (e.g., concentrated breeding) also have an important effect on the intra-annual variation in the dynamics of *C. burnetii*. Naturally acquired humoral immunity seems to have no effect on future re-infection of deer by *C. burnetii*, perhaps linked to the observed short average half-life of antibodies in red deer.

## Author Contributions

DG-B and FR-F designed the study and wrote the manuscript; DG-B, IF-M, JO, JQ, and FR-F collected samples; DG-B performed serological analyses; DG-B and FR-F analyzed data; DG-B, IF-M, JO, JQ, and FR-F critically reviewed the manuscript.

## Conflict of Interest Statement

The authors declare that the research was conducted in the absence of any commercial or financial relationships that could be considered as a potential conflict of interest.
